# The roles of patient‐derived xenograft models and artificial intelligence toward precision medicine

**DOI:** 10.1002/mco2.745

**Published:** 2024-09-25

**Authors:** Venkatachalababu Janitri, Kandasamy Nagarajan ArulJothi, Vijay Murali Ravi Mythili, Sachin Kumar Singh, Parteek Prasher, Gaurav Gupta, Kamal Dua, Rakshith Hanumanthappa, Karthikeyan Karthikeyan, Krishnan Anand

**Affiliations:** ^1^ Department of Biomedical Engineering Rochester Institute of Technology Rochester New York USA; ^2^ Department of Genetic Engineering, College of Engineering and Technology SRM Institute of Science and Technology Chengalpattu Tamil Nadu India; ^3^ School of Pharmaceutical Sciences Lovely Professional University Phagwara Punjab India; ^4^ Department of Chemistry University of Petroleum & Energy Studies, Energy Acres Dehradun India; ^5^ Centre for Research Impact & Outcome, Chitkara College of Pharmacy Chitkara University Rajpura Punjab India; ^6^ Faculty of Health, Australian Research Center in Complementary and Integrative, Medicine University of Technology Sydney Ultimo NSW Australia; ^7^ Discipline of Pharmacy, Graduate School of Health University of Technology Sydney Ultimo NSW Australia; ^8^ JSS Banashankari Arts, Commerce, and SK Gubbi Science College Karnatak University Dharwad Karnataka India; ^9^ Centre of Excellence in PCB Design and Analysis, Department of Electronics and Communication Engineering M. Kumarasamy College of Engineering Karur Tamil Nadu India; ^10^ Department of Chemical Pathology, School of Pathology, Office of the Dean, Faculty of Health Sciences University of the Free State Bloemfontein South Africa

**Keywords:** artificial intelligence, cancer biology, nanodrug delivery, patient‐derived xenografts, PDX model, personalized medicine, tumor genetics, tumor modeling

## Abstract

Patient‐derived xenografts (PDX) involve transplanting patient cells or tissues into immunodeficient mice, offering superior disease models compared with cell line xenografts and genetically engineered mice. In contrast to traditional cell‐line xenografts and genetically engineered mice, PDX models harbor the molecular and biologic features from the original patient tumor and are generationally stable. This high fidelity makes PDX models particularly suitable for preclinical and coclinical drug testing, therefore better predicting therapeutic efficacy. Although PDX models are becoming more useful, the several factors influencing their reliability and predictive power are not well understood. Several existing studies have looked into the possibility that PDX models could be important in enhancing our knowledge with regard to tumor genetics, biomarker discovery, and personalized medicine; however, a number of problems still need to be addressed, such as the high cost and time‐consuming processes involved, together with the variability in tumor take rates. This review addresses these gaps by detailing the methodologies to generate PDX models, their application in cancer research, and their advantages over other models. Further, it elaborates on how artificial intelligence and machine learning were incorporated into PDX studies to fast‐track therapeutic evaluation. This review is an overview of the progress that has been done so far in using PDX models for cancer research and shows their potential to be further improved in improving our understanding of oncogenesis.

## Graphical abstract

The PDX models revolutionize clinical research. PDX models, obtained from patient‐derived cells, are one of the latest tools in translational medicine and represent very close models of human tumor biology. This can be combined with the PDX model through advanced technologies such as artificial intelligence, machine learning, 3D/4D bioprinting, and organ‐on‐a‐chip systems to rapidly accelerate therapeutic evaluation. AI and ML improve in silico predictive analytics by identification of effective drug responses, while 3D/4D bioprinting and organ‐on‐a‐chip platforms provide sophisticated dynamic microenvironments that very closely recapitulate human physiology. Convergence of these technologies dramatically accelerates the development of personalized treatments, ensuring more precise and effective therapeutic interventions (Created using BioRender).

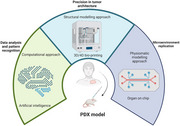

## 1 INTRODUCTION

The ability to accurately represent human diseases is critical to biomedical research. In this endeavor, animal models have proven to be excellent instruments for dissecting complicated biological processes and assessing therapeutic approaches.^1^ From Alcmaeon of Croton's pioneering studies on canine intelligence to the current rush to create COVID‐19 vaccines, animal models have played an important part in innovations that have considerably improved human and animal health.^2^ The serendipitous intervention of the state of the art scientific and technological realities of 21st century, namely, artificial intelligence (AI), machine learning (ML), deep learning (DL), organ‐on‐chips systems (OOC), 3D and 4D bioprinting, omics techniques, and so on, has provided us with a fresh paradigm in terms of utilizing animal models for medical research.^3^

In the olden days, scientific experiments were carried out either in wild or domesticated animals, whereas the urge for refined data and the complexity of newly developed diseases and conditions warranted animal models of specific nature. This led to the generation of customized animal models to meet the precise requirements of the research problem. Indeed, the modern research era has witnessed the advent of genetically identical, genetically engineered or reprogrammed, immunodeficient, patient‐derived xenograft (PDX) and humanized PDX animal models for various research requirements.^4^

This review aims to discuss the roads that led to the generation of PDX models, the methods employed to develop PDX models, their applications in basic and translational research, role of AI in PDX models, advantages, limitations, and challenges associated with them. PDX are the models of a disease where the cells or tissues from a patient are entrenched into an immunodeficient mouse. The history of PDXs goes back to the 1970s when Rygaard and Poulsen developed the first PDX mouse model from the tumor excised from a 74‐year‐old colonic adenocarcinoma patient by injecting the minced tissue subcutaneously in nude mice.^5^ Multiple studies carried out in the 1980s, investigated the chemotherapeutic responses in PDX models, and the results correlated with the responses observed in patients of the tumor origin. PDX models, due to their “humanized” feature, were involved in preclinical studies and clinical trials to test novel compounds.^6^ Furthermore, the primary xenograft model derived from small cell lung carcinoma (SCLC) exhibited a similar gene expression pattern to that of the patient's SCLC tumor sample that substantiates the validity of the PDX model for basic and translational research.^7^

In the past decade, the PDX model has excelled to become an invaluable asset to the cancer research community for its umpteen applications, which include tumor genetics, biomarker discovery, metastatic progression studies, personalized therapy, and above all for minutely mimicking the in vivo cancer biology.^8^, ^9^, ^10^, ^11^, ^12^ This examination will delve further into the construction and deployment of PDX models. We embark on a tour through the history of PDX technology and its status in cancer research. Furthermore, this review will offer light on the emerging role of AI and ML in optimizing PDX development and analysis, enabling a new degree of efficiency and accuracy. Finally, we will demonstrate how PDX models can be used in a variety of ways to advance basic, translational, and personalized cancer research. This study aims to provide a critical and current perspective on the enormous promise of PDX models as we work to overcome the difficult obstacle of cancer.

## 2 METHODS OF GENERATING PDX MODELS

**FIGURE 1:**
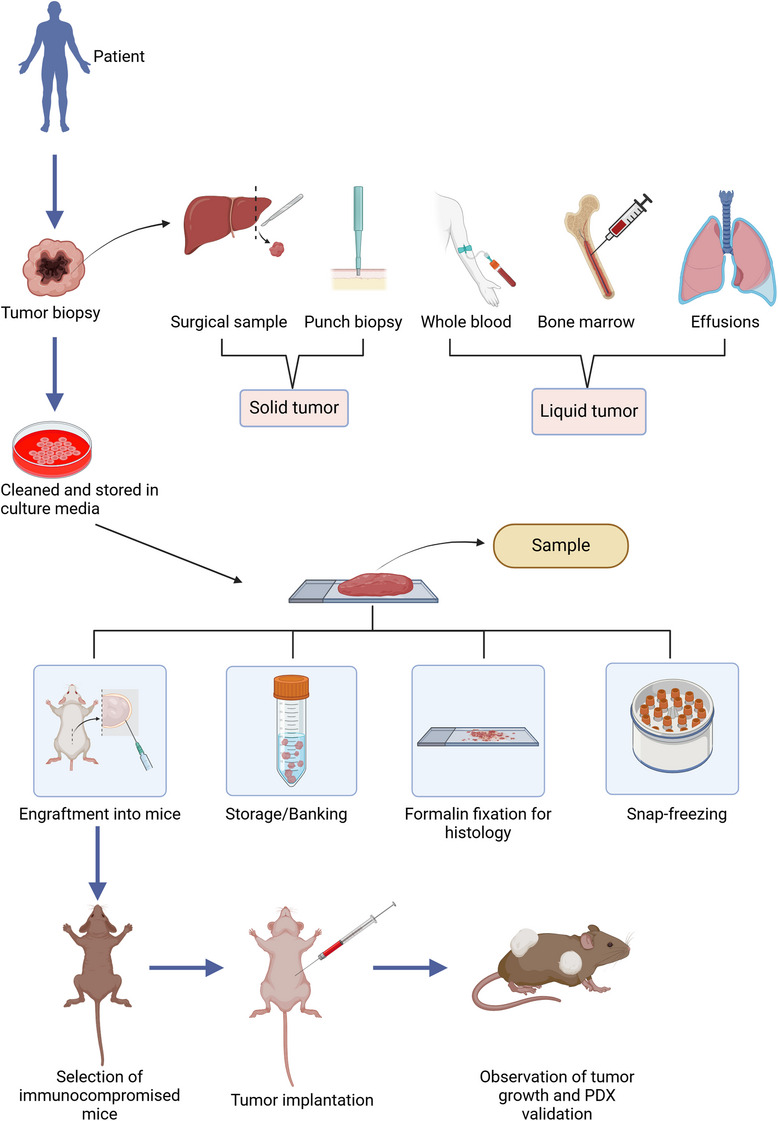
Schematic illustration of the overall process involved in the generation of PDX models. The tumor biopsy recovery methods for different types of tumors are shown here. The recovered samples will be subjected for further processing before engraftment. The processed tumor will be validated with an appropriate method, followed by engraftment in animal model. Some portion of the tissue/cells will be stored for further use (Created using BioRender).

**FIGURE 2:**
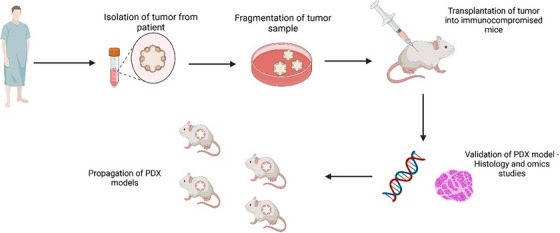
Specific procedure involved in grafting to establish PDX model. The validation of patient derived xenograft in mouse models is done through acquisition of patient tumor and further propagation of PDX models for the development of preclinical research and individualized treatment strategies. (Created using BioRender).

The establishment of PDX models is a laborious process, as it is not only time consuming but also requires quite a lot of funding and personnel.^13^ The correct coordination between clinicians, surgeons, and researchers is of paramount importance as it involves time‐sensitive steps that can affect the fate of the success rate of the PDX model.^14^ A typical workflow for establishing a PDX model as depicted in Figure 1 is to select the patients’ fulfilling the specific criteria and obtain consent as per the Institutional Review Board protocol. A schematic representation of the procedure is described in Figure 2. Upon obtaining the tumor sample through the right medical procedure from the clinician, the sample must be transported to the research facility swiftly.^14^ The researchers must keep themselves ready with all the required materials and reagents and on receiving the tissue, must process it and implant it in the mice and/or must store it for further analysis. The model thus developed is characterized on a molecular and histopathological basis and confirmed with the parent tumor.^15^, ^16^, ^17^ The final process is proper sample annotation and documentation for each step during the establishment of PDX modeling.^18^

### 2.1 Assessment and preparation of tumor tissue

Screening is done to identify potential tumor specimens for PDX development since all cancer patients cannot be sampled.^10^ Only study interest and hypothesis drive sample screening.^19^ Researchers usually select the tumor for which there are no current therapies available or for which there is no existing model to study.^20^ Some laboratories focus on generating PDX libraries to better understand the heterogeneity and genomic characteristics of particular cancer and its subtypes.^21^, ^22^, ^23^ Libraries can also be generated by creating models from the same patients throughout their disease course, which provide insight into tumor progression and mechanism/mode of resistance.^24^ The sample collection is a very tedious process that involves coordinated efforts from clinical to clerical staff. The sample collection must be performed aseptically under all circumstances to avoid contamination in cell culture or during engraftment of the sample in mice.^25^ The sample can either be solid or liquid. It is better to choose noninvasive tissue acquisition like core needle biopsies, for which the lesion size should be in the range of 1.5−2 cm to allow for at least 2 cores of 10 mm each in length.^26^ A typical punch biopsy produces a 3−4 mm cylindrical tissue core, which is often used for obtaining cutaneous tumor tissues.^27^ Endoscopic procedures are widely used for gastrointestinal tract cancers from which 2−3 mm^3^ cores can be procured.^28^, ^29^ In the case of hematological malignancies, a minimum of 5 mL of noncoagulated peripheral blood or bone marrow aspirate would be required to generate a sufficient number of mononuclear cells (MNC). The solid tumor tissue is collected in culture media (RPMI or DMEM), saline (0.9% sodium chloride), antibiotics, and antimycotics. In cases where the samples cannot be processed immediately, storing the samples to maintain tissue viability is paramount. Hypothermsol™ can be used to preserve tumor cell viability for up to 48 h. The sample from the autopsy should be retrieved within 8−12 h from the time of death as there will be a rapid loss of cell viability post‐mortem.^30^ The liquid samples from pleural or pericardial effusions, bone marrow aspirates, and ascites contain viable cells, which have shown better take rates in comparison with solid tumor engraftment. The samples are immediately treated with heparin (1 mL heparin/liter of fluid) to avoid clotting and for easy maneuvering while processing. Whole blood (leukemia and circulating tumor cells CTCs) and bone marrow are directly collected in anticoagulant coated tubes.^31^ The sample once collected is extremely precious and used wisely and to its maximum.^32^ When there is an ample amount of tissue, it can be fixed in 10% buffered formalin or 4% paraformaldehyde or an equivalent fixative for IHC (immunohistochemistry) analysis, snap frozen in liquid nitrogen for genomics or biochemical assays.^33^, ^34^, ^35^ The tissue can be processed into a single‐cell suspension and cryopreserved or viably freezing as an intact tissue. For leukemia samples, it is necessary to process the blood to obtain MNC for viable storage of cells that are further treated for cell purifications or direct engraftment. It is highly recommended to collect a normal tissue specimen alongside the tumor sample for comparative analysis and negative controls.^33^, ^34^, ^35^, ^36^ In the case of nonhematological cancers, blood is usually considered a normal tissue specimen, and for germline DNA, hematological malignancies, buccal swabs, and fingernail clippings are used as normal tissue specimens. For DNA sequencing, blood or buccal samples would be ideal; however, for transcriptomics, a nontumorigenic part of the same tissue should be used as the mRNA expression is highly tissue specific. The tumors obtained via surgeries are usually heterogenous and need thorough processing to remove the necrotic part of the tumor and any cysts to increase the take rate of tumor in mice.^37^ For the initial processing of the whole tumor tissue, surgeons or pathologists may help with and handover the tumorigenic part of the tissue to the technician in the collection media. The viable content of the tumor region contains both tumor cells and stromal components, which will have a different color, morphology, shape, and consistency, usually less opaque and firmer in comparison with the adjoining healthy tissue.^38^ Necrotic tissue is quite distinguishable because of its location mostly being in the center of huge tumors. The appearance of necrotic tissue ranges from opaque or white to very dark or blackish color and concerning the texture, it can be brittle or as hard as a rock or in a liquid state. The stromal component is distinct being translucent and elastic in nature.^39^ The healthy tumor is vascularized and appears pink or reddish in color, which is selected and cut into 3 mm^3^ in size.^40^, ^41^, ^42^ The fragments are prepared to be used for injection into mice, for banking, snap frozen to perform genomic analysis, and are formalin‐fixed to perform histology. The size of the tissue will tell us if all the above‐mentioned usages can be done with the tissue obtained. The surgically isolated sample or biopsies that are <1 g is usually implanted without any further processing to increase its take rate and larger fragments are cut into 2−3 mm^3^ sections and implanted.^43^ Despite the cons of single‐cell suspension, there seem to be more advantages linked to it, the tumor cell viability is easily accessed and specific subpopulations of a heterogeneous tumor type can be studied by targeted isolation and cell implantation into mice.^43^ The added advantage is that single‐cell suspensions can be injected via subcutaneous (SC) and orthotopic (OT) and they can be cultured in vitro to generate novel cell lines. The processing of the liquid sample is entirely different from how solid tumors are processed. In hematological tumors, samples are liquid‐like blood and bone marrow aspirates from which MNCs are isolated by Ficoll density gradient centrifugation, followed by red blood cells (RBCs) lysis for implantation into mice to generate PDX models. In highly metastatic tumors, the circulating tumor cells (CTCs) can be isolated that are found in the blood stream that can be isolated from peripheral blood.^31^ The isolation of CTCs is enriched using commercially available special enrichment buffers that help in the selection of tumor cells. The CTCs and tumor cells are implanted into the mice using IV injections.^44^, ^45^ The samples from procedures like effusions and ascites collection are directly subjected to centrifugation, for isolating the tumor cells, the cell pellet obtained from centrifugation is treated with RBC lysis buffer multiple times and the resulting cell population is injected into the mice via SC or orthotopic (OT).

### 2.2 Validation of established PDX model

The success of the generation of the PDX model is certified only after verifying the genomic, molecular, and histopathological characteristics of the established tumor with the parent or patient tumor.^46^ To verify the genomic profile of mice tumor and its human counterpart various techniques from DNA sequencing to species‐specific and gene‐specific PCRs are done.^47^ Sequencing data show the evolutionary path of recurrent tumors, explaining therapy failure and tumor resistance. The tumor patients’ normal tissue negative control is crucial for distinguishing germline and somatic variations. Multiple platforms like whole genome sequencing, exome sequencing, transcriptome sequencing, single‐cell RNA sequencing, and targeted sequencing assays like MSK‐IMPACT can capture genomic data, so a database is needed to consolidate and analyze the data and provide comparative cross‐species and longitudinal data analysis.^23^, ^48^, ^49^ The patient's tissue, F0 generation, and serially transplanted existing generation like F1, F2, F3, and so on must be routinely processed for H and E staining and reviewed by a board‐certified pathologist to confirm that the lesions match and correspond to the tumor of interest. Staining is necessary to rule out nonspecific lesions caused by an inflammatory reaction at the implantation site, unrelated tumors, or spontaneous tumors of host or human origin.^50^ While host tumors need treatment, inflammatory reactions are frequently granulomas or abscesses and can be separated from them. Although most mouse tumors are lymphomas, fusiform cell sarcomas and mammary gland tumors have been identified. As indicated, the EBV infects cells at the implantation site, causing nonlymphoid lymphomas. B cells can be removed in immunocompetent mice but develop cancerous in immunocompromised mice.^51^, ^52^ After histological inspection, tumor cells must be differentiated to determine their ability to differentiate throughout successive passagings. Serial passaging can cause the tumor to lose its ability to differentiate like the parent tumor and change morphologically and genetically. Due to tumor heterogeneity and host characteristics, distinct PDX models from the same specimen will have different F0 generations.^53^ The human and PDX specimens’ histology should reflect the universal histological pattern for the tumor of interest, and particular biomarkers must be checked across specimens. Whatever the case, all created PDXs must be histopathologically analyzed every few passages and H and E staining and other IHC slides must be stored and noted for future reference and review.^48^, ^54^

## 3 APPLICATIONS OF PDX MODELS

PDX models are superior to both, cell line xenografts and genetically engineered mouse models. In theory, the generation and characterization of PDX models that retain critical molecular and biological properties of their tumor of origin as well as represent the full spectrum of heterogeneity of various cancers would represent an exceptionally powerful tool for translational research.^55^ This is especially important in the context of pre‐ and coclinical studies, as the predictive potential of PDX models will aid in effective drug selection.^56^, ^57^ The potential power of PDXs is based on the fact that they are biologically stable. If they are maintained in vivo by directly passaging from mouse to mouse, their characteristics will closely resemble the primary tumor for several generations. Therefore, PDX models can be biologically and molecularly investigated at a greater depth than any given patient sample, allowing for a better understanding of the molecular mechanisms governing oncogenesis.^58^ Furthermore, as they are in theory an unlimited resource, PDXs can be challenged with numerous candidate therapeutics, or treatment regimens, in a relatively short time frame that cannot be accomplished in the clinic (Table 1). Herein, we will briefly discuss the broad applications of the PDX model in translational research in both molecular and drug‐based investigations.

**TABLE 1:** Selected examples of therapeutic strategies assessed using PDX models with details of the cancer type, specific target(s), and mouse models used.

Cancer type	Drug or combination	Target	Mouse	References
Acute myelogenous leukemia	CSL362 monoclonal antibody with cytarabine and daunorubicin	CD123	NSG	^71^
Acute myelogenous leukemia	Brequinar	Dihydroorotate dehydrogenase	SCID	^72^
Acute myelogenous leukemia	R406	Syk	NOG	^73^
Acute myelogenous leukemia	Selinexor (KPT‐330)	XPO1	NSG	^74^
Multiple myeloma	BI‐505 antibody	ICAM‐1	SCID	^75^
B cell acute lymphoblastic leukemias	CHZ868 (JAK2 inhibitor) and dexamethasone	JAK2	NSG	^76^
BRAF mutant cancer	PD0325901	MEK	Nude	^77^
Breast carcinoma	Dinaciclib	CDK	NOD/SCID	^78^
Breast carcinoma	FRAX597 (PAK2 inhibitor) and fulvestrant	PAK2 and ER	NSG	^41^
Breast carcinoma	ICG‐001 (Wnt inhibitor) and doxorubicin	Wnt signaling	NSG	^79^
Breast carcinoma	decitabine	DNA methyltransferases	NOD/SCID	^80^
Cholangiocarcinoma	Ponatinib, dovitinib, and BGJ398	FGFR	NSG	^81^
Chordomas	Erlotinib and gefitinib	EGFR	Nude	^82^
Colon cancer	Cetuximab and pimasertib	EGFR/MEK	NOD/SCID	^83^
Colon cancer	FP3	VEGF	Nude	^84^
Colon cancer	Cetuximab and bevacizumab	EGFR/VEGF	Nude	^85^
Colorectal cancer	DEL‐22379	Erk	NOD/SCID	^86^
Colorectal cancer	Anti‐RSPO3 (antibody)	RSPO3	Nude	^87^
Esophageal squamous cell carcinoma	Trastuzumab	Her2	Nude and SCID	^88^
Gastric cancer	Luteolin	cMet	Nude	^89^
Gastric cancer	Trastuzumab and cetuximab	Her2/EGFR	Nude	^81^
Gastric cancer	AZD5363	AKT	Nude	^90^
Lung adenocarcinoma	Cetuximab	EGFR	NOD/SCID	^91^
Lung squamous cell carcinoma	BGJ398 and cisplatin	AKT and ERK	NSG	^92^
Lung cancer	Erlotinib and thalidomide	EGFR, TNF, and NF‐kB	NOD/SCID	^93^
Lung cancer	BDA‐366	Bcl2 BH4 domain	Nude	^94^
Lymphoma	Pyruvinium pamoate	Glutathione	NOG	^95^, ^96^
Medulloblastoma	Fingolimod (FTY720)	–	Nude	^96^
Melanoma	Salmonella A1‐R	–	Nude	^97^
Melanoma	Karonudib (TH1579)	MTH1	NOG	^98^
Melanoma	Vemurafenib and fatostatin	SREBP‐1	Nude	^99^
Melanoma	TH287 and TH588	MTH1	NOG	^100^
Multiple cancers	CFI‐400945, inhibitor	PLK4	NSG and SCID	^101^
Melanoma	CCT196969, CCT241F161	pan‐RAF and SFKs	Nude	^102^
Multiple myeloma	P5091	USP7	SCID	^103^
Multiple myeloma and solid tumors	CB‐5083	p97	Nude and SCID‐Beige	^26^
Neuroblastoma	MLN8237 and ABT‐199	Aurora kinase and BCL‐2	SCID	^104^
Non‐Hodgkin lymphoma	Anti‐CD47 antibody and rituximab	CD47	NSG	^105^
Chronic myelogenous leukemia	NSC23766	Rac	NOD/SCID	^106^
Pancreatic adenocarcinoma	BKM120	PI3K inhibitor	NSG	^107^
Pancreatic cancer	IGF1‐IONP‐Dox	IGF1R	Nude mice/SCID	^108^
Pancreatic cancer	Trametinib and dasatinib	MEK/Tyrosine kinase Src	NSG	^109^
Pancreatic cancer	Phenformin	Mitochondrial complex I	Nude	^110^
Prostate cancer	EPI‐001	Androgen receptor NTD domain	NOD/SCID	^111^
Prostate cancer	Bicalutamide	Androgen	SCID	^112^
Renal cell carcinoma	PT2399	HIF‐2	Nude	^113^
Sarcoma	Salmonella A1‐R and doxorubicin	–	Nude	^114^
Small cell lung cancer	GSK2879552	LSD1	Nude	^115^
Pancreatic cancer	Trametinib and MRTX1133	KRAS	Nude	^116^
Mesothelioma	Gemcitabine	_	Nude	^117^
Pancreatic cancer	Gemcitabine, 5‐fluorouracil	_	Nude	^118^
Mesonephric adenocarcinoma	Paclitaxel, cisplatin	_	Nude	^119^

### 3.1 Molecular applications of the PDX model

The principle of molecular cancer research is to unravel the complexity associated with the origins and progression of cancer. This improved understanding allows for improved prediction and ultimately, better therapeutics to treat a wide range of cancers.^59^ In this regard, the PDX model is a great tool as it can be used to generate both in vivo and ex vivo data and some of the most common uses of this model are summarized below.

#### 3.1.1 Interrogation of clonal evolution

Intratumoral heterogeneity is common in solid tumors due to the evolution of genetically diverse subclones.^60^, ^61^ In this regard, PDX models are ideal tools as closely mimic human cancers and this allows for the investigation of the molecular, cellular and subclonal characteristics of various cancer types.^62^, ^63^ For example, using a PDX model it has been shown that a minor cell subpopulation is capable to drive tumor growth. This subpopulation had enhanced proliferative ability and was capable to overcome the microenvironmental constraints when compared with other cells within the tumor.^64^, ^65^ In another study, acute lymphoblastic leukemia PDXs were used to identify a small population of unfavorable dormant cells that were treatment resistant and could accurately mimic the patients’ primary cells.^66^, ^67^ In acute myeloid leukemia PDX models, the relationship between clonal architecture and functional heterogeneity was investigated and it was shown that there was variable engraftment potential, with the successful xenografts predominantly comprising a single genetically defined subclone.^67^, ^68^ Last, a basal‐like primary breast cancer tumor, a PDX model derived from this tumor, and the resulting brain metastasis from a patient was assessed using deep genomic analyses. Interestingly, when compared with the primary tumor, the brain metastatic sample contained de novo mutations and deletions.^69^ Furthermore, while the PDX retained the mutations present in the primary tumor, it was genetically similar to the brain metastasis sample.^70^

#### 3.1.2 Cancer cell initiation, proliferation, and drug resistance

High‐throughput genome sequencing has identified countless somatic mutations in cancers; however, there is a poor understanding of the functional impact of many of these mutations.^120^ In this regard, PDX models can be used to determine which specific mutations have a direct impact on tumor formation and those that confer resistance to therapy.^121^ Cancer cell proliferation is a common hallmark assessed to determine the effect of a specific mutation^122^; however, it is important to note that cancer cells in a PDX model do not follow the standard linear growth rate and instead have an exponential dynamic growth rate that increases over time. Importantly, this information has direct implications for the interpretation of translational studies.^123^ Furthermore, the identification of key cellular mechanisms involved in cancer cell initiation and proliferation has benefitted from the use of PDX models.^124^ This is seen in the context of cancer stem cells (CSCs), also known as tumor initiating cells (TICs). CSCs are a small neoplastic cell population with stem cell properties that can perpetuate themselves via auto‐restoration and are considered a major cause of therapeutic resistance.^125^, ^126^ Indeed, it has been shown in a xenograft model that CD133^+^ human brain TICs were able to initiate tumors in vivo, which provided insight into human brain tumor pathogenesis as well as supporting the neoplastic role of CSC in solid tumors.^127^, ^128^ Traditionally, genetically modified cancer cell line xenograft models are used to validate in vitro results of the intrinsic molecular mechanisms involved in tumorigenesis.^129^ However, this model is not adequate as it does not accurately recapitulate malignancy and in vitro genetic manipulation and expansion of primary tumor cells are challenging. These challenges can be overcome by using PDX models and the functional significance of this is seen by investigations of microRNAs (miRNAs). miRNAs are widely recognized as important post‐transcriptional regulators of gene expression and a B‐Cell Acute Lymphoblastic Leukemia PDX model was used to investigate miRNA‐126.^130^ It was shown that miRNA‐126 played a key role in cancer progression by targeting p53‐dependent pathways leading to the evasion of cell‐cycle arrest and apoptosis. Last, it was seen that antagonizing miRNA‐126 in human patient samples was sufficient to reduce the disease burden in its PDX model by triggering apoptosis.^131^

### 3.2 Preclinical cancer research uses of the PDX model

Historically, the use of immortalized cell lines has been the cornerstone of preclinical cancer research.^132^ However, this model system has been underwhelming in its ability to evaluate the heterogeneity in a patient's tumor or to successfully identify novel therapeutics. Indeed, this is evident as many investigational phase III studies of anticancer drugs with positive tumor responses in mouse models, do not correlate with clinical trials in patients ultimately resulting in failure.^133^, ^134^ As stated earlier, PDX models retain the heterogeneity of patient tumors, allowing for investigating the efficacy of therapies. While PDX models are versatile tools in preclinical research the main reported uses will be discussed in the following sections.

#### 3.2.1 Identification of cancer biomarkers

Cancer‐specific biomarkers are an important component of cancer research as they can aid in successful diagnosis and assessing the prognosis of specific cancer, and in some cases, identify novel and effective therapeutic targets.^135^ The impact of the PDX model has been shown by Bradford et al.^136^ when they performed whole‐transcriptome profiling of 79 PDX models across a range of different cancer types. They aimed to identify independent tumor and stromal biomarkers and using this information, explore the interaction between these two compartments. Indeed, they reported a potential interaction between two hypoxia‐associated genes, human MIF and mouse Ddx6. Interestingly, it has been established that the efficacy of anticancer drugs is influenced by the tumor–stroma interaction, and this novel use of the PDX model has the potential to improve preclinical drug efficacy studies by further exploring resistance mechanisms. The prognostic value of the stem cell markers, CD133 and CD44, has been investigated in PDX models of cancers such as hepatocellular carcinoma.^137^, ^138^ In addition, in a bladder cancer PDX, the overexpression of cell division cycle 25C (CDC25C) has been shown to be a predictive biomarker and is therefore a novel molecular target.^139^ Interestingly, Gardner et al.^140^ generated small‐cell lung cancer PDX models that were paired chemo‐naive and chemo resistant. Using this model, they reported that EZH2 was a biomarker of chemoresistance as EZH2 was able to epigenetically silence SLFN11.^140^ Recently, it has been shown using triple negative breast cancer PDX models that DNA methyltransferase is an effective predictive biomarker of the efficacy of the United States Food and Drug Administration (US FDA)‐approved drug decitabine and further highlights the importance of identifying biomarkers predictive of therapeutic response, which will allow for improved patient care and prognosis.^80^

#### 3.2.2 Investigation of experimental anticancer therapeutic approaches

PDX models are useful in investigating and establishing experimental anticancer therapeutic approaches.^141^ For example, a study has investigated using trans‐differentiation‐derived induced neural stem cells as a therapeutic intervention in glioblastoma PDX models.^142^ These cells were genetically engineered to contain both optical reporters and tumoricidal gene products and were found to target glioblastoma cancer cells. In addition, when this methodology was used to deliver the anticancer drug, TRAIL, there was decreased growth of glioblastoma PDX models.^143^ The benefit of the pancreatic cancer PDX models allowed for preclinical evaluation of precise fluorescence‐guided surgery (FGS), which can significantly aid in surgery and therefore improve the outcome for patients with recalcitrant cancers.^144^ This technique can attach different fluorescent colors to cancer and stroma cells, respectively, therefore allowing for their identification and complete resection including stroma. Therefore, this method significantly prevented local recurrence, which the standard bright‐light surgery or single‐color FGS could not.^145^ In addition, this method was further evaluated in a PDX model of colon cancer with fluorophore‐conjugated anti‐CEA antibody.^146^, ^147^ Interestingly, a pancreatic cancer PDX model was utilized to investigate the efficacy of an IGF1 receptor‐directed nanoparticle conjugated to the chemotherapeutic drug, Doxorubicin. This was a novel approach as stromal barriers and the TME play a role in poor drug delivery and this approach takes advantage of the fact that IGF1R is highly expressed in both pancreatic cancer cells and stromal fibroblasts.^108^ In another study, a head and neck squamous cell carcinoma PDX model was used to evaluate the potent anticancer effects of encapsulating a PI3Kα inhibitor, BYL719, into P‐selectin‐targeted nanoparticles. Importantly, results showed that the treatment resulted in significant inhibitions of tumor growth and increased radio‐sensitization at a dose that was seven‐fold lower than oral administration. Last, radiation studies using PDXs have been limited in their scope and number when compared with chemotherapeutic agent studies, as reviewed by^148^

#### 3.2.3 Evaluation of anticancer therapeutic strategies

There is currently a high attrition rate in the field of anticancer agents with only 5% being approved by the US FDA despite promising preclinical anticancer effects. It has therefore been suggested that the current models for drug testing (cell line xenograft or genetically engineered mouse) fail to capture the effects of tumor heterogeneity as well as the influence of the human stromal microenvironment leading to high failure rate.^149^ It is therefore unsurprising that preclinical drug testing is one of the most well‐described uses of PDX models. These models allow for an early indication of drug safety, efficacy, and evaluation of an appropriate treatment dosage before moving into an animal model. Indeed, PDX model‐based anticancer drug development in specific cancers has been discussed comprehensively.^38^, ^150^, ^151^, ^152^, ^153^ Here, we give a summary of the preclinical uses of the PDX model, and a summary of representative drugs, or drug combinations and their targets are shown.

#### 3.2.4 Combinational approaches

The idea of combined targeting of two or more onco‐signalling pathways is a promising strategy for cancer therapy.^154^ The rationale is that specific targeted cancer therapies often lead to the selection of resistant populations. Therefore, by selecting therapies with differing but complimentary mechanisms of action and combining them, the resistant populations can be reduced.^155^ Indeed, multiple studies have used PDX models to show the value of this strategy. For example, the combination of disulfiram, a drug used to treat alcoholism, and copper was assessed for its efficacy against B‐ALL. In the study, the authors found that the treatment reduced tumor cell growth while sparing normal peripheral blood MNCs.^35^ Another example is the use of CDK4/6 inhibitors that can resensitize PDX tumors to HER2‐targeted therapies and delay tumor recurrence.^156^ Similar results have been seen with the Aurora kinase A inhibitor MLN8237 and ABT‐199 acting synergistically to counter MYCN‐amplified neuroblastomas PDX models.^104^ The combination of CDK4/6‐PI3K inhibition in PIK3CA mutant breast cancer PDXs has also shown promise.^157^ The anti‐CD47 antibody in combination with rituximab was synergistic and led to the promotion of phagocytosis, which allowed for the elimination of lymphoma in both disseminated and localized non‐Hodgkin lymphoma PDX models.^105^ Last, it was found that chemo‐resistant triple‐negative breast cancer‐derived PDX's expressed high levels of Wnt10B related molecules. When the combination of ICG‐001, a Wnt signaling inhibitor, and doxorubicin were administered in the PDX model there was efficient repression of lung dissemination as a result of shedding from the original shedding.^79^, ^158^

### 3.3 Coclinical trials and precision medicine

**FIGURE 3:**
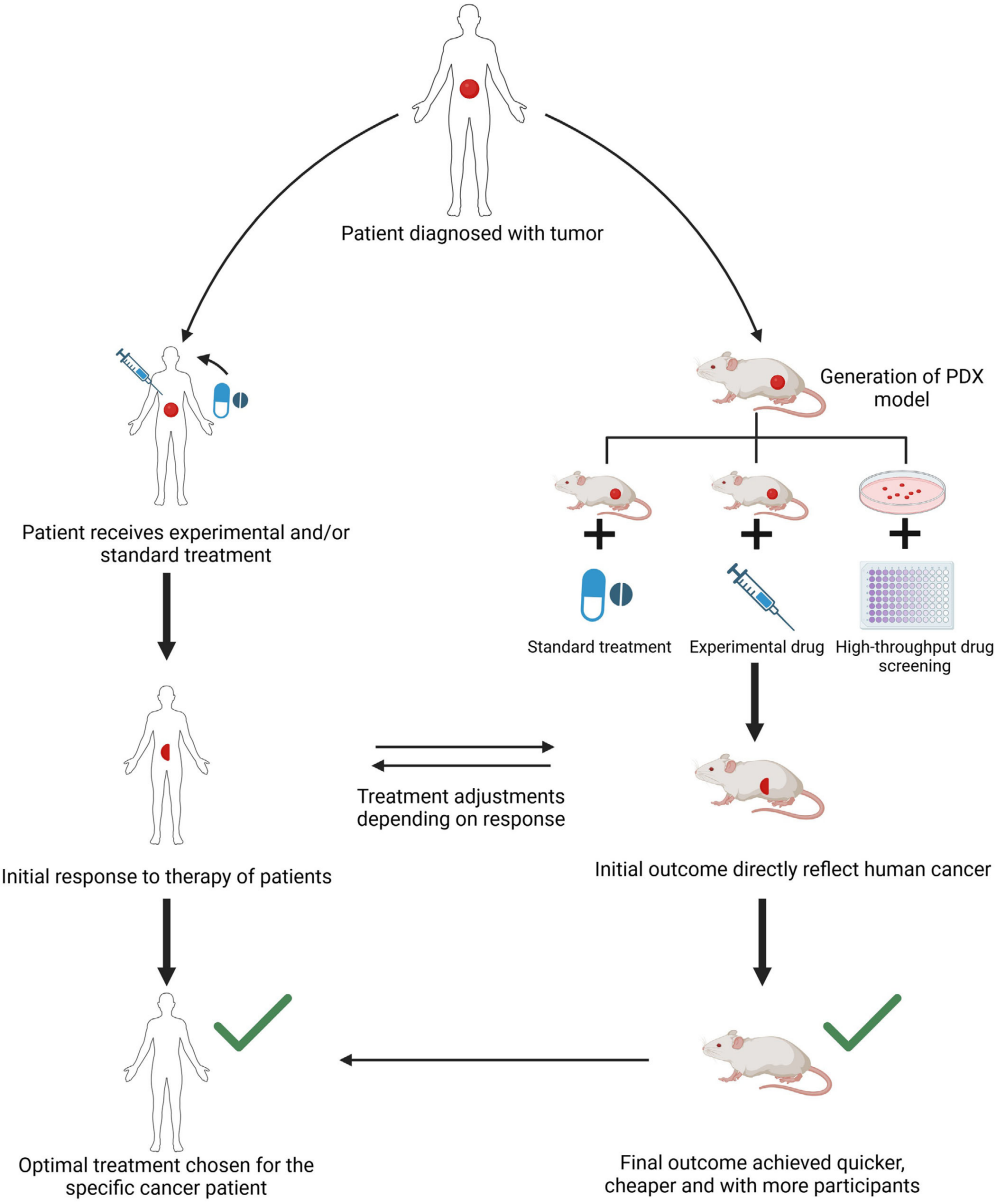
Schematic representation involved in coclinical trial workflow for precision medicine. The PDX model thus generated is further processed for developing precision medicine and personalized care that involves integrating preclinical studies followed by clinical trials. This approach enables the identification of biomarkers and genetic profiles to predict patient responses.

The most compelling data highlighting the power of the PDX model is in predicting the potential benefits of both conventional and novel anticancer therapeutics for cancer patients. As mentioned previously, the high failure rate in cancer drug development is a major issue as this consumes a considerable number of resources with very little public benefit.^159^ The current preclinical drug screening models have poor predictive potential as they do not select specific patients or fully capture the complexity seen in patient tumor samples. The PDX model is a useful tool in this regard as it is theoretically an unlimited source of patient tumor sample with the potential to be expanded and manipulated in vivo and ex vivo.^160^ Several studies have used the PDX model to study drug responses in several cancers and have seen that there is a high level of correlation between the drug response rates in PDX models and patients in the clinic.^161^, ^162^, ^163^ For example, clinical patient data and the renal cell cancer PDX model matched as they showed positive responses to sirolimus, sunitinib, and dovitinib while showing no response to erlotinib.^164^, ^165^ Indeed, conventional chemotherapy studies in several cancers, including breast, colorectal, and pancreatic cancer, have also shown the same trend in which the PDX model and clinical data had comparable responses to the standard clinical chemotherapy agents such as paclitaxel, carboplatin, gemcitabine, and adriamycin.^166^, ^167^ This has led to suggestions that PDX models could be employed in “co‐clinical trials” in which a preclinical trial can be conducted on a mouse PDX model in parallel with a patient undergoing a clinical trial treatment.^168^ Therefore, this workflow allows for the incorporation of patient selection strategies based on molecular abnormalities and the identification of novel biomarkers of sensitivity or resistance to anticancer agents^169^ (Figure 3). Based on this, novel combination strategies can be proposed. For example, Heid et al.^170^ performed a coclinical assessment of tumor cellularity in pancreatic cancer. Owonikoko et al.^171^ also reported that PDX faithfully replicated clinical outcomes in phase II coclinical trial of arsenic trioxide in relapsed small‐cell lung cancer.

## 4 ADVANTAGES OF PDX MODELS

PDX models have prominent advantages in pre‐ and coclinical studies compared with the genetically engineered models and cell line‐derived xenograft models. The specific advantages of PDX models are discussed in the following sections.

### 4.1 Applying genetic engineering methods

Genetic engineering techniques such as gene knock‐out and knock‐in strategies are used to introduce the tumorigenic factors in CDX and GEMs and it is worth noting that, it is highly unlikely to recreate the entire set of factors in the model organisms.^172^ The PDX models circumvent the use of genetic engineering techniques since they contain most of the factors in vivo required for tumorigenesis.

### 4.2 Microenvironment

Before the advent of PDX models, monolayer cell culture, spheroids, and organoids were used in basic and translational research.^173^ Although these models generate a considerate amount of data, they still lack the microenvironment of the tumor and interaction with the adjacent tissues/organs, which might skew the behavior of the cells and treatment responses.^174^, ^175^ Hence, a seamless model is expected to possess the maximum characteristic features of an in vivo environment. The animal models (PDX, CDX, and GEM) were preferred over the aforesaid models, due to the simulation of a similar microenvironment.^25^, ^176^, ^177^ A PDX model is considered more meritorious, as they contain cells from the actual tumor from the patients and mimic the tumor environment in vivo with a complete set of oncogenic elements such as hypoxia, blood supply, heterogeneity of tumor cell subpopulation, and the extracellular matrix.^178^

### 4.3 Gene expression pattern

Gene expression pattern decides the behavioral pattern of the cell or tissue. Since the PDX models are expected to mimic the human tumor microenvironment, it is important to speculate the gene expression patterns between the cancerous tissue from the patient and the tumor developed in the PDX model. It has been shown that the gene expression pattern in primary SCLC and the xenograft model were identical, while the expression of tumor‐specific genes is lost in the xenograft‐derived cell lines.^7^ Thus, the usage of PDX model organisms with matched gene expression pattern is expected to give precise predictive outcomes in translational research.^7^

### 4.4 Patient‐oriented response

The pre‐ and coclinical studies are aimed to obtain results that match patient‐oriented responses in clinical trials. It is alarming that for cancer drugs, the results obtained with cell culture models can not accurately predict the therapeutic efficacy.^149^ PDX models are reported to provide outcomes that are highly similar to those of the patients.^21^

## 5 CHALLENGES AND LIMITATIONS OF PDX MODELS

Although PDX models have evolved with an exciting opportunity for improving the values that are predicted in preclinical and translational studies, some several limitations and challenges need to be addressed to improve their use in the medical field. Overcoming these issues could increase the potentiality of the model that will increase therapeutic applications.^48^, ^179^

### 5.1 Immuno‐challenged models lack immune cells

In all the tumor types, immune cells play a vital role in tumor progression and growth.^180^, ^181^ PDX models chiefly depend on the murine immunodeficiency models that lack functional elements of immune systems. Therefore, the cancer cells cannot reproduce the interaction between cancer cells and immune cells that exist in the patient tumor. Thus, it makes the model highly challenging as it leads to difficulty in predicting the efficacy of the drug and also to analyze the mechanism of drug resistance.^9^, ^182^, ^183^, ^184^ This lacuna was well documented in tumors such as melanoma where the treatment has been done by targeting immunotherapy.^185^, ^186^

### 5.2 Take rates are low

The take‐up rates of transplanted tumors differ from that of the tumors of origin.^187^ Currently, the statistical analysis reported that the take‐up rates of the patient‐derived breast cancers are very low although it is found to be enhanced take‐up rates due to the development of pre‐exposed methods of estrogen in luminal type tumors.^188^

Additionally, such low take‐up rates and the long‐term incubation time in the transplanted mice are current challenges faced by researchers in using the PDX models for pre‐ or coclinical studies. Although there are technical advances that have improved the take rates, different types of tumors and their subtypes within the same tumor type have various success rates. This leads to the imbalanced representation of tumor types that are more determined by take rate than clinical incident rate. The limitations of such problems of PDX models might be resolved by the development of suitable mice for PDX models or any suitable methods of tumor transplantation that enhance the take rates.^189^ Another major limitation of the PDX model is that there are chances that the tumors fail to progress or to metastasize and thus do not retain all the disease patterns observed in patients. To overcome this issue, patient‐derived orthotopic xenografts represent a powerful tool to address the key point in preclinical modeling.

### 5.3 Problems with the sampling size

Initially, the most appropriate tissue is vital to transplant into the mice model and if the tumor size is larger, one part of it should be taken for the PDX study. In this step, there is a need for certified pathologists and other experts, which might be a major limitation. Additionally, the tissue extracted must be analyzed immediately for the efficient generation of PDX.^190^ In most cases, there is a requirement for smaller size samples such as fine needle aspirations necessary for transplantation for the application of personalized medicine. Thus, there is a limitation in studying the PDX technique in a small specimen and thus should be developed in this aspect.

### 5.4 Strategy for engraftment

Another challenge in using the PDX model is that there should be a defined strategy for engraftment in mice, based on the tumor types. In general, OT implantation has been followed these days, but again special technique in the surgical aspect is required based on the cancer type and this takes a lot of time and effort. Of course, subcutaneous implantation can be considered as it has a little higher success rate with a simple procedure, but they do not corroborate with the comparable microenvironment of the primary tumor.^191^

### 5.5 Duration of treatment

Another challenge faced by the technologists is the duration of survival and treatment schedules of the patients that are the major criteria that must be satisfied in this current scenario in the application of personalized medicine. The normal developing time of the PDX model is from 2 to 8 months for any preclinical study, which is again an extensive time for the patient to wait. And the duration is the major limiting factor for individualized medicine. These again might involve some failures and be considered the success rates while personalization that might still take some time for the patient. Thus, discovering the suitable conditions for subtypes of cancer might elevate the duration of PDX generation.^192^

### 5.6 Mouse–human engraftment ratio

The key aspect in the PDX research is to make use of the mouse models that are an immune deficient strain for any kind of engraftment and propagation as the mouse should avoid rejection of the human tissue.^193^ For the same reason, the research has avoided using the conventional type of PDX model that involves the screening with agents such as vaccines and checkpoints that blocks the antibodies of the host system, and rather utilizing the humanized PDX model that helps in transplanting the human hematopoietic stem cells, which is the alternative option.^41^, ^194^ Murine fibroblasts differ from that of humans,^195^ which is why we need a successful mouse model for the study.^41^

### 5.7 High cost

Of course, while everything is on one side, the financial aspect will stand single on the other side. To take into consideration the financial cost of the PDX model, highly immunodeficient mice are very expensive. Additionally, maintaining those mice in a clean environment also takes a high cost, as the maintenance might take a long time until the tumors are engrafted and PDX models are developed.^152^ Another factor to consider is that cloned animal and genome sequencing analysis costs a lot and experimental preclinical expenses are also high enough to proceed with the PDX models. So not all patients might be able to afford these costs and hence PDXs remains technically challenging and also time consuming.^41^, ^196^, ^197^

## 6 ROLE OF MODERN TECHNOLOGIES IN PDX MODELS

### 6.1 Artificial intelligence(AI)

**FIGURE 4:**
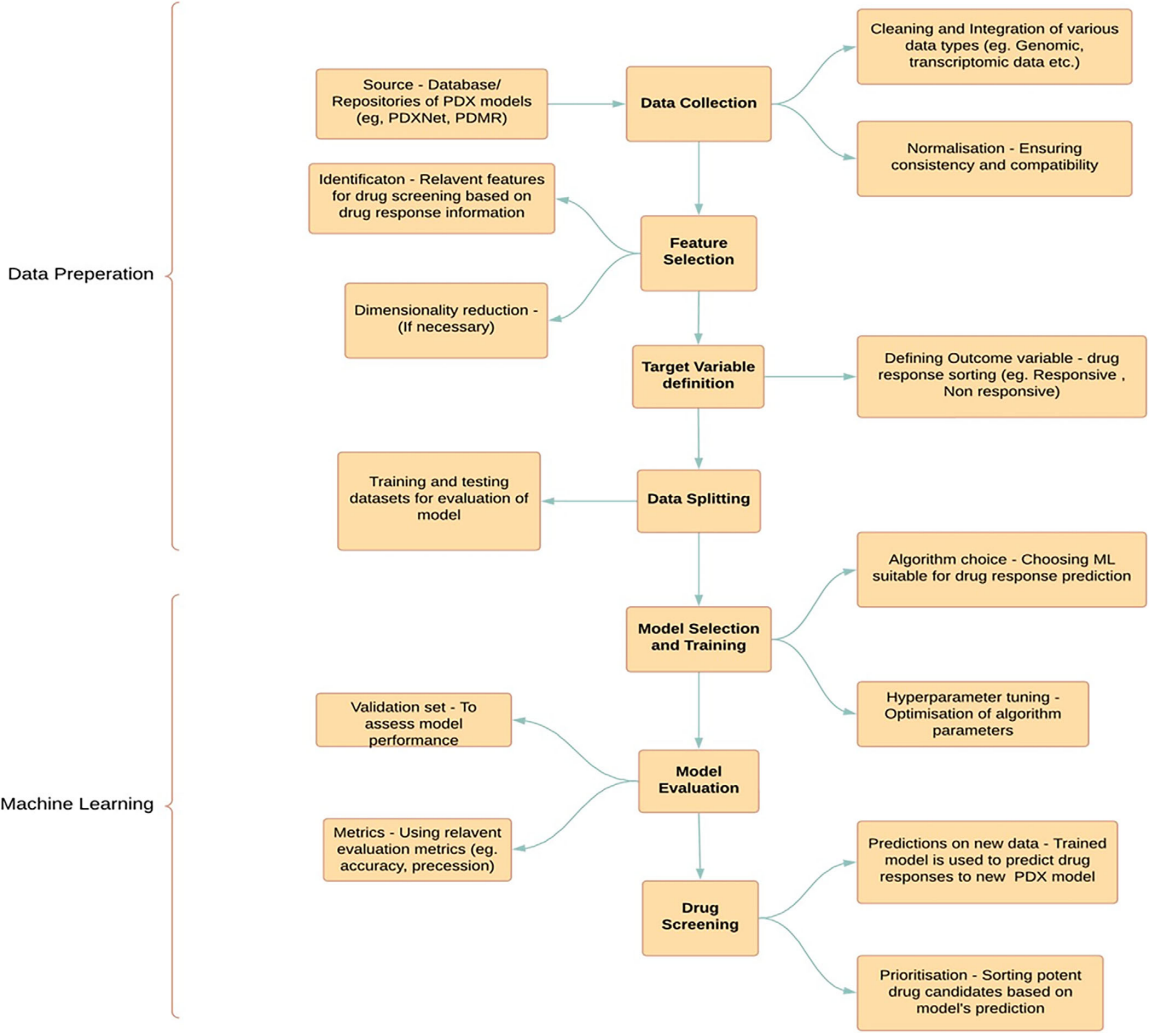
Process chart describing the workflow for screening of drugs using Machine Learning. The machine learning workflow for drug screening using patient‐derived xenograft (PDX) models involves training algorithms on large datasets derived from PDX models to predict drug responses.

The integration of AI with PDX models is critical due to PDX models' great ability to recapitulate tumor heterogeneity and treatment responses.^198^, ^199^ Researchers can better comprehend tumor evolution and medication responses by integrating AI and PDX models as depicted in Figure 4. AI can also help in identifying and removing contaminated host sequences in PDX models, confirming the accuracy of sequencing data and mutation calls.^200^, ^201^ Furthermore, the integration of AI in PDX models can assist personalized therapy by allowing exact monitoring of carcinogenesis and biophysical tumor features in real‐time, potentially leading to advances in individualized treatment methods and improved patient outcomes.^202^ Additionally, AI has been used to predict pathological complete response in hormone receptor‐positive/human epidermal growth factor receptor 2‐negative breast cancer patients following neoadjuvant chemotherapy, assisting doctors in personalizing treatment plans.^198^, ^203^ Several AI methods have been proposed to extract human‐centered rule‐sets from black‐box models such as neural networks (NNs), allowing for a better understanding of their decision‐making processes. The use of AI in pancreatic ductal adenocarcinoma has also expanded, particularly in organ segmentation, AI‐aided diagnosis, and radiomics‐based personalized treatment, demonstrating the promise for earlier detection and better decision‐making in this aggressive tumor.^204^

### 6.2 Machine Learning (ML) and Deep learning (DL)

**FIGURE 5:**
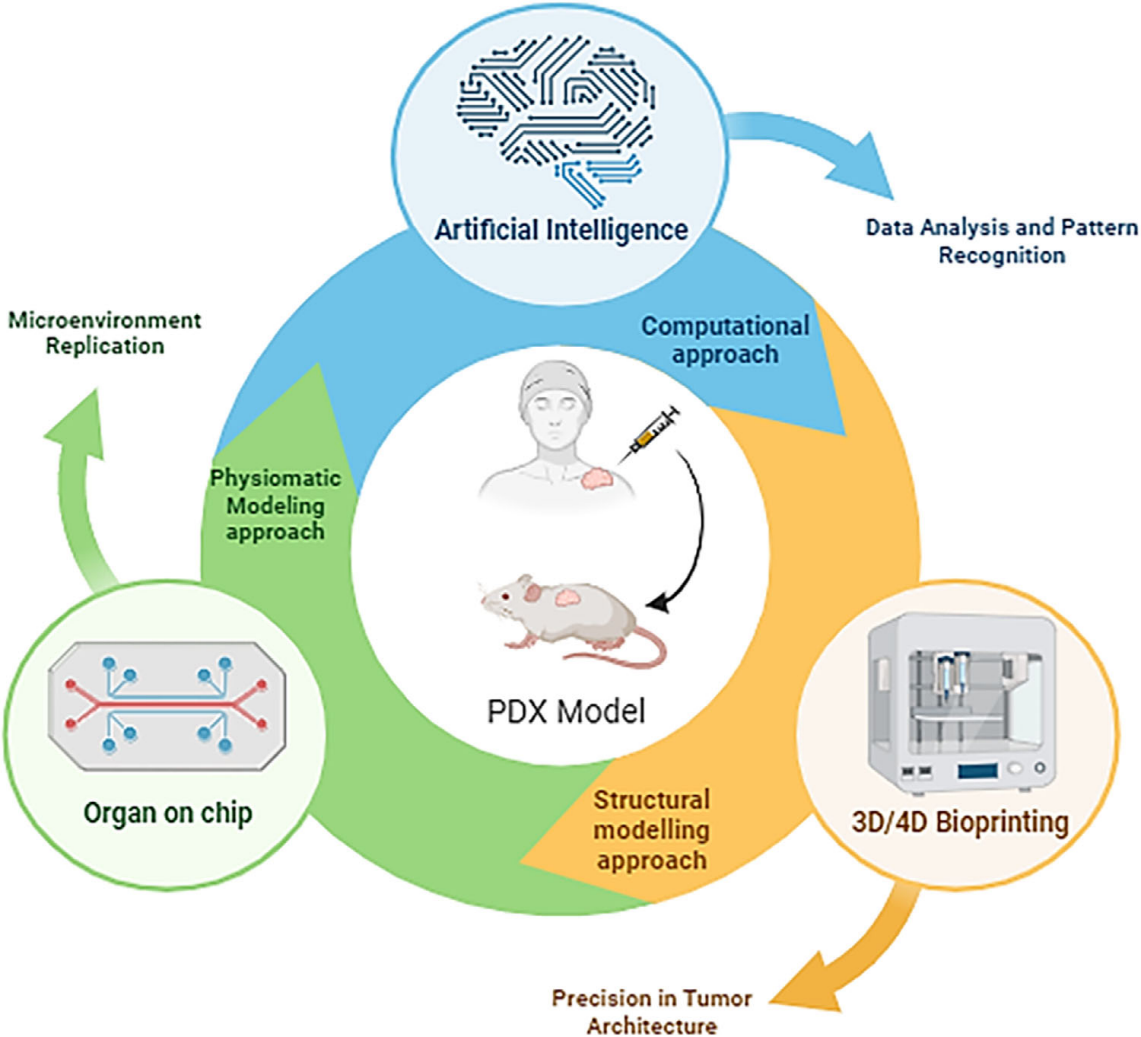
Pictorial representation of integrating advanced technologies in the validation of PDX models. Integrating advanced technologies in PDX modeling offers a holistic approach by combining genomic, transcriptomic, and proteomic data to enhance the accuracy of tumor biology representation. This integration allows for better prediction of therapeutic responses, personalized treatment strategies, and accelerated drug development through real‐time data analysis and multiomics approaches. (Created using BioRender).

The impact of ever evolving advanced technologies on hottest biomedical research avenues like cancer treatment and precession medicine is immeasurable and is gaining momentum at a pace that was never expected before. Specifically, the new age techniques of 3D bioprinting, AI, that is, ML and DL have been subjected to thorough investigations to assess their applicability to construct computational models for anticancer drug response predictions and many more (Figure 5).^205^, ^206^ Employing such vigorous tools paves the way for exploring novel perspectives for cognizing and characterizing potential drug candidates for personalized cancer therapies. PDX models are proven to be super‐efficient tools and immensely used in the domain of cancer research as they mimic the heterogeneity of the actual tumors.^207^, ^208^ Thus, becoming ideal experimental models and also can significantly contribute to unraveling the intricacies of tumor multifariousness, which is a fundamental factor influencing the treatment outcomes.^209^, ^210^ Various patients with the same cancer type can express distinct molecular profiles and responses to the given treatments. In addition to that, precision medicine emphasizes the significance of customizing treatments based on the specific genetic, molecular, and environmental factors affecting an individual's disorder. PDX models, with their ability to withhold the distinctive attributes of patient tumors provide a powerful platform for testing the efficacy of different treatment regimens in a personalized manner. Since these models necessitate grafting of human tumor tissues into immunodeficient mice that can facilitate various drug screening studies of pharmacological importance, it also can aid in understanding the biology of tumors, elucidating drug resistance mechanisms, evaluating and exploring new therapies, and so on. The field of preclinical and coclinical testing is currently undergoing a revolutionary transformation a result of the integration of throughput technologies like ML and DL in PDX model‐based studies. The role of databases and repositories is instrumental in terms of model building and processing in the domain of ML and DL. These specialized and organized collections comprise of plethora of dataset that is served as the substrate for the learning objectives of the computational algorithms.^211^ In ML, assembling the datasets (both input and relevant output labels) is the foremost step that forms the fundamental base line source that is used to train ML algorithms in which it recognizes complex patterns and relationships present in the data. Thus, formed ML model is capable to prognosticate the similar and unperceived data. DL is a sub‐branch of ML that specifically flourishes on large‐scale datasets for training complex NNs. Eventually, the nature, multiplicity and range of databases impact the efficacy of ML and DL models, underlining the indispensable role of data in the dynamic realm of AI. Gao et al., Koc et al., and Kim et al.^212^, ^213^, ^214^, ^215^ have used the various comprehensive PDX‐based databases and repositories, namely, Novartis Institutes for Biomedical Research PDX Encyclopedia (NIBR PDXE), National Cancer Institute (NCI) patient‐derived models repository (PDMR), PDX network (PDXNet) Portal, and so on, to train ML models and to explore various objectives aiding in development of efficient AI‐driven models in precession medicine.

#### 6.2.1 The NIBR PDXE

This extensive repository established by Novartis houses an extensive collection of over 1000 PDX models. More than 1000 PDX models have been established.^212^ It can be employed in preclinical cancer drug development and stands as a revolutionary enterprise in the landscape of precision oncology.^216^

#### 6.2.2 The NCI patient—PDMR

This repository was developed by the NCI, USA, which consists of in vitro PDXs, patient‐derived tumor cell cultures and cancer‐associated fibroblasts as well as patient‐derived organoids. This collection can be attributed as an important asset majorly aiming for the application in quantification of targets, pharmacodynamic assays, predictive marker development, and clinical trials for future research in drug discovery.^217^

#### 6.2.3 PDXNet portal

This particular database aims to empower collaborative research by open sourcing the data and ensuring the easy accessibility among scientific and nonscientific community. Currently this portal comprises of resources from 334 new models across 33 cancer type. It is updated regularly and efficiently applied to carry out studies on multiagent treatment, decoding resistance mechanisms, sensitivity determination, and preclinical trials.^213^, ^218^

Optimal model complexity (OMC), which refers to the critical balance between model simplicity and performance in ML, is another key technical strategy that is considered, especially in the context of PDX modeling. In order to achieve the OMC, the model must be optimized such that it is complex enough to detect unapparent trends in the PDX data without being overly intricate and over fitting to noise or discrepancies in the training set. A model is said to be over fit when it learns the training set very well, including the noise/distortion in it, which results in poor generalization on fresh data. On the other hand, inadequate modeling occurs when a model is overly simplistic or underperform to encompass the intricacies in the PDX data leading to less‐than‐ideal predicted accuracy. Locating the OMC usually requires calibrating the hyper parameters, evaluating performance on validation datasets and conducting repetitive experiments with diverse model complexities. Achieving the proper balance guarantees that the ML model broadly applies to various patient‐specific experiences and hence enhancing the reliability and precision of predictions in medication response and other biological contexts. Individual treatments rules (ITRs) also known as personalized treatment rules are a set of clinical recommendations based on the patient profiles in order to maximize the efficacy of the given treatment by personalizing it according to a subpopulation or an individual since patients show differential responses for the drugs,^219^, ^220^ is yet another vertical of precision oncology wherein PDX models and associated data clubbed with ML can also be extrapolated to quantify them further proving the potency and flexibility of the application of ML in precision oncology.

### 6.3 Cancer Risk Prediction Model Knowledge Base database and its relation with PDX models

The Cancer Risk Prediction Model Knowledge Base (CRPMKB) plays a crucial role in providing a centralized platform for storing and comparing cancer risk prediction models. It contains detailed information on 802 model data, allowing researchers to systematically compare the accuracy of cancer risk predictions based on regional differences, cancer types, and model types. CRPMKB categorizes model variables into environment, behavioral lifestyle, biological genetics, and clinical examination, highlighting the differences in variable distribution among different cancer types. By conducting pathway enrichment analyses on genes involved in specific cancer risk prediction models, such as lung cancer, CRPMKB helps identify significant pathways like p53 signaling and aryl hydrocarbon receptor signaling, aiding in understanding the biological mechanisms underlying cancer development. Researchers can utilize CRPMKB for personalized model applications and development, enhancing the accuracy of cancer risk predictions by creating more targeted models based on specific demographic characteristics and cancer types. The platform also offers functionalities like data display, retrieval, submission, information sharing, and platform management, making it a comprehensive tool for data‐driven research and personalized applications of cancer risk prediction models.^221^ PDX models, which implant patient tumor tissues into immunodeficient mice, can be combined with CRPMKB models to improve cancer research and personalized therapy. By adding PDX model data into CRPMKB, researchers may test and improve cancer risk prediction models based on experimental results and real‐world tumor behavior. The integration of PDX model data with CRPMKB provides a more thorough understanding of cancer biology, allowing for the discovery of new biomarkers and treatment targets for specific cancer types. Researchers can use data from PDX models to test the predictive accuracy of CRPMKB models, resulting in the creation of more precise and personalized cancer risk assessment systems. The link between PDX models and CRPMKB accelerates translational research by bridging the gap between preclinical studies and clinical applications, ultimately enhancing patient outcomes in cancer diagnosis and treatment.

### 6.4 OOC and 3D / 4D bioprinting

The applications of PDX models illustrate huge leap in the domain of cancer research in the light of OOC systems and 3D/4D bioprinting technologies. OOC systems are engineered microscale devices to emulate the structure and functionality of specific organs contributing to an environment that closely resembles essential physiological traits. OOCs provide an organ's microenvironment to examine tumor–stroma interactions, medication responses and cancer progression. These novel techniques aim to act in synergy with the conventional in vitro research and in vivo impediments. When used with PDX models, more realistic representation of in vivo circumstances is facilitated by OOC systems, which mimic the milieu of certain organs including the tumor niche, blood arteries, and extracellular matrix. According to Huh et al.,^222^ one example of a device that can simulate the respiratory environment is a lung‐on‐a‐chip. This innovative approach allows researchers to examine lung metastasis and how the body reacts to treatment in an environment that is very similar to humans.^222^ Simultaneously, advancements in 3D and 4D bioprinting technologies have a major impact on improving the way in which tumor microenvironment is represented in PDX models. The translational ability of traditional 2D cell cultures is hindered since they often fail to encapsulate the stereographic complexity of malignancies. In order to develop spatially defined and physiologically relevant tissue architectures, 3D bioprinting entails the layer‐by‐layer deposition of bioinks comprising cells, growth factors and biomaterials.^223^, ^224^ It also allows the creation of multicellular tumor spheroids or organoids in a biomimetic extracellular matrix for use with Parkinson's disease PDX models.^225^ This method implies to meticulous representation of the cellular heterogeneity and tumor architecture. In addition to that, by using stimuli‐responsive materials that permits dynamic changes over time 4D bioprinting an improved form of its predecessor 3D bioprinting introduces the temporal dimension with which, tissue constructions may be created in a regulated artificial environment that replicates the aggressive processes of tumor development and response to therapy^226^, ^227^ A dynamic change in approaches for cancer research has taken place with the combination of 3D/4D bioprinting technologies and OOC systems with PDX models. The probability and possibility of discovering previously unseen aspects of personalized medicine is becoming more and clearer as we continue to explore and exploit the potential of these technologies.^228^

## 7 FUTURE DIRECTIONS

The interest in using the PDX model in cancer research applications has been growing.^150^, ^229^ From the literature cited, it is evident that this model system has been important in progressing various fields; however, there are additional research areas that need to be improved to further this exciting research model, for example, in the field of implantation success rates. Currently, numerous research groups are expanding the research on the PDX model; however, there is a lack of standardization amongst them concerning minimum sample size, preservation media, additional supplements to aid engraftment, and the site of engraftment. Despite these challenges, there have been numerous success stories but this field of translational research will greatly benefit if the ability to engraft all, including difficult‐to‐engraft tumors such as prostate cancer, could be standardized and successful protocols could be shared amongst research groups. The use of this model system in high‐throughput drug screening is extremely attractive especially with the potential for ex vivo manipulation there is a significant chance of modifications to the fundamental and unique biological properties of the patient‐specific tumor, thus negating the translational value of the PDX model.^7^, ^230^ Interestingly, while it is well established that the PDX model represents a genetically heterogeneous model at any given time, it will only be able to provide a snapshot of a single time point of this highly complex disease. Therefore, each PDX model may not be able to fully represent the complex nature of cancers. In addition, the most successful PDX models are the forms of most aggressive phenotypes of this disease, while this is counterintuitive, this is an interesting avenue to pursue as these tumors are the most resistant to therapy and are the ones most in need of novel therapeutic models as they represent end‐stage disease.^231^, ^232^ As stated earlier, the most significant benefit of this model is in its ability to accurately predict the efficacy of therapeutic interventions and therefore it needs to be incorporated into the drug design pipeline. While tumor regression is the desired endpoint for most treatments, it is important to recognize that different drugs, for example, anti‐CSC drugs, may have different desired endpoints, for instance, growth delay and latency to resistance development. Overall, the advantages of the PDX model, such as its ability to retain tumor characteristics provide it with many advantages in preclinical tests of drug screening, biomarker development, and coclinical trials.^162^, ^233^ The advent and usage of advanced AI/ML technologies, OOC, 3D/4D bioprinting and omics‐based tools such as NGS, allow for a comprehensive analysis of complex data, visualization of tumor structure, reconstruction, and so on, thus, comes out as a promising avenue in biomedical research.^234^, ^235^, ^236^ We believe the PDX model is one of the appropriate and exciting preclinical tools to widen the field of personalized medicine as well as understand the complex field of cancer.^45^, ^237^

## AUTHOR CONTRIBUTIONS

*Conceptualization*: Kandasamy Nagarajan ArulJothi, Krishnan Anand, Gaurav Gupta, and Kamal Dua. *Material collection, ideation, and analysis*: Venkatachalababu Janitri, Karthikeyan Karthikeyan, VijayMurali Ravi Mythili, and Sachin Kumar Singh. *Writing the original draft*: Venkatachalababu Janitri, Kandasamy Nagarajan ArulJothi, VijayMurali Ravi Mythili, Rakshith Hanumanthappa, Karthikeyan Karthikeyan, Parteek Prasher, and Sachin Kumar Singh. *Review and editing*: Kandasamy Nagarajan ArulJothi, Krishnan Anand, Kamal Dua, and Karthikeyan Karthikeyan. All authors have read and approved the final manuscript.

## CONFLICT OF INTEREST STATEMENT

The authors declare that there are no conflict of interest.

## ETHICS STATEMENT

No animals or human samples were used in the study.

## Data Availability

There are no raw data used in this manuscript.
